# Supplementary material for: Hybrid Models and Biological Model Reduction with PyDSTool
Source: PLoS Comput Biol. 2012 Aug 9;8(8):e1002628. doi: 10.1371/journal.pcbi.1002628 (PMC3415397; doi:10.1371/journal.pcbi.1002628)
Supplement: Text S4 — Complete source code for the PyDSTool package (version 0.88.120504). Includes API documentation and help files linking to web pages. This file is identical to the current public release on Sourceforge.net. (ZIP) [file pcbi.1002628.s004.zip › PyDSTool/html/PyDSTool.Symbolic.Quantity-class.html]

xml version="1.0" encoding="ascii"?


PyDSTool.Symbolic.Quantity


| Home | Trees | Indices | Help | PyDSTool |
| --- | --- | --- | --- | --- |

Package PyDSTool ::
Module Symbolic ::
Class Quantity

[hide private]

[frames] | no frames]

# Class Quantity

source code

```
object --+
         |
        Quantity
```

Known Subclasses:
:   - Fun
    - , Input
    - , Par
    - , Var

---

Abstract class for symbolic numerical quantities.


|  |  |
| --- | --- |
| Instance Methods | [hide private] |

|  |  |
| --- | --- |
| \_\_init\_\_(self, spec, name=`'``'`, domain=None, specType=None)  x.\_\_init\_\_(...) initializes x; see x.\_\_class\_\_.\_\_doc\_\_ for signature | source code |

|  |  |
| --- | --- |
| setDomain(self, domain) | source code |

|  |  |
| --- | --- |
| redefine(self, specStr) | source code |

|  |  |
| --- | --- |
| isspecdefined(self) | source code |

|  |  |
| --- | --- |
| \_\_contains\_\_(self, tok) | source code |

|  |  |
| --- | --- |
| \_\_call\_\_(self) | source code |

|  |  |
| --- | --- |
| \_\_len\_\_(self) | source code |

|  |  |
| --- | --- |
| isvector(self)  Is the quantity spec of the form [a,b,c] | source code |

|  |  |
| --- | --- |
| fromvector(self, ix=None)  Turn the Quantity's specification of a vector to a list of the element's specifications. | source code |

|  |  |
| --- | --- |
| \_\_setitem\_\_(self, x, v) | source code |

|  |  |
| --- | --- |
| \_\_delitem\_\_(self, x) | source code |

|  |  |
| --- | --- |
| \_\_getitem\_\_(self, x) | source code |

|  |  |
| --- | --- |
| tonumeric(self) | source code |

|  |  |
| --- | --- |
| \_\_combine(self, other, opstr, reverseorder=0) | source code |

|  |  |
| --- | --- |
| \_\_add\_\_(self, other) | source code |

|  |  |
| --- | --- |
| \_\_mul\_\_(self, other) | source code |

|  |  |
| --- | --- |
| \_\_sub\_\_(self, other) | source code |

|  |  |
| --- | --- |
| \_\_div\_\_(self, other) | source code |

|  |  |
| --- | --- |
| \_\_radd\_\_(self, other) | source code |

|  |  |
| --- | --- |
| \_\_rmul\_\_(self, other) | source code |

|  |  |
| --- | --- |
| \_\_rsub\_\_(self, other) | source code |

|  |  |
| --- | --- |
| \_\_rdiv\_\_(self, other) | source code |

|  |  |
| --- | --- |
| \_\_truediv\_\_(self, other) | source code |

|  |  |
| --- | --- |
| \_\_rtruediv\_\_(self, other) | source code |

|  |  |
| --- | --- |
| \_\_pow\_\_(self, other) | source code |

|  |  |
| --- | --- |
| \_\_rpow\_\_(self, other) | source code |

|  |  |
| --- | --- |
| \_\_neg\_\_(self) | source code |

|  |  |
| --- | --- |
| \_\_pos\_\_(self) | source code |

|  |  |
| --- | --- |
| mapNames(self, nameMap) | source code |

|  |  |
| --- | --- |
| compileFuncSpec(self) | source code |

|  |  |
| --- | --- |
| simplify(self) | source code |

|  |  |
| --- | --- |
| renderForCode(self)  Return code-ready version of spec (e.g. | source code |

|  |  |
| --- | --- |
| rename(self, new\_name) | source code |

|  |  |
| --- | --- |
| eval(self, \*scopearg, \*\*defs)  eval does not require all free symbols to be resolved, but any signature arguments (for a function) must be resolved. | source code |

|  |  |
| --- | --- |
| validate(self) | source code |

|  |  |
| --- | --- |
| isDefined(self, verbose=False) | source code |

|  |  |
| --- | --- |
| \_\_repr\_\_(self)  repr(x) | source code |

|  |  |
| --- | --- |
| \_\_str\_\_(self)  str(x) | source code |

|  |  |
| --- | --- |
| info(self, verboselevel=1) | source code |

|  |  |
| --- | --- |
| \_\_hash\_\_(self)  hash(x) | source code |

|  |  |
| --- | --- |
| \_\_eq\_\_(self, other, diff=False) | source code |

|  |  |
| --- | --- |
| \_\_ne\_\_(self, other) | source code |

|  |  |
| --- | --- |
| difference(self, other)  Show differences in comparison to another Quantity. | source code |

|  |  |
| --- | --- |
| \_\_copy\_\_(self) | source code |

|  |  |
| --- | --- |
| \_\_deepcopy\_\_(self, memo=None, \_nil=`[``]`) | source code |

**Inherited from `object`**:
`__delattr__`,
`__getattribute__`,
`__new__`,
`__reduce__`,
`__reduce_ex__`,
`__setattr__`


|  |  |
| --- | --- |
| Class Variables | [hide private] |

typestr = `None`

compatibleGens = `()`

targetLangs = `()`


|  |  |
| --- | --- |
| Properties | [hide private] |

**Inherited from `object`**:
`__class__`


|  |  |
| --- | --- |
| Method Details | [hide private] |

|  |  |
| --- | --- |
| \_\_init\_\_(self, spec, name=`'``'`, domain=None, specType=None)  *(Constructor)* | source code |

x.\_\_init\_\_(...) initializes x; see x.\_\_class\_\_.\_\_doc\_\_ for
signature

Overrides: object.\_\_init\_\_: *(inherited documentation)*

|  |  |
| --- | --- |
| renderForCode(self) | source code |

Return code-ready version of spec (e.g. uncapitalize built-in function
names)

|  |  |
| --- | --- |
| \_\_repr\_\_(self)  *(Representation operator)* | source code |

repr(x)

Overrides: object.\_\_repr\_\_: *(inherited documentation)*

|  |  |
| --- | --- |
| \_\_str\_\_(self)  *(Informal representation operator)* | source code |

str(x)

Overrides: object.\_\_str\_\_: *(inherited documentation)*

|  |  |
| --- | --- |
| \_\_hash\_\_(self)  *(Hashing function)* | source code |

hash(x)

Overrides: object.\_\_hash\_\_: *(inherited documentation)*

  


| Home | Trees | Indices | Help | PyDSTool |
| --- | --- | --- | --- | --- |

|  |  |
| --- | --- |
| Generated by Epydoc 3.0.1 on Fri May 4 15:24:08 2012 | http://epydoc.sourceforge.net |
